# Treatment effect of posterior scleral reinforcement on controlling myopia progression: A systematic review and meta-analysis

**DOI:** 10.1371/journal.pone.0233564

**Published:** 2020-05-26

**Authors:** Chih-An Chen, Pao-Yen Lin, Pei-Chang Wu

**Affiliations:** 1 Department of Ophthalmology, Kaohsiung Chang Gung Memorial Hospital and Chang Gung University College of Medicine, Kaohsiung, Taiwan; 2 Department of Psychiatry, Kaohsiung Chang Gung Memorial Hospital and Chang Gung University College of Medicine, Kaohsiung, Taiwan; National Yang-Ming University Hospital, TAIWAN

## Abstract

**Background:**

High myopia is a sight-threatening disease that causes axial length elongation and severe complications. Data on the benefits of posterior scleral reinforcement surgery in myopia control have been conflicting. The purpose of this study was to explore the treatment effect and complications of posterior scleral reinforcement in the treatment of myopia.

**Methods:**

Articles were retrieved for relevant studies from inception to July 24, 2019, by PubMed, EMBASE, and Ovid. Analyses were conducted to compare the treatment effects of controlling spherical equivalent refraction and axial length elongation. The weighted mean difference and Hedges’ adjusted *g* were used to evaluate the treatment effects, with a random-effects model. Heterogeneity was quantified using *I*^2^ statistic and explored by subgroup analysis. Publication bias was addressed by funnel plots and Egger’s test.

**Results:**

A total of 11 articles were included in this meta-analysis. On estimating the treatment effect, the mean differences of myopia progression and axial length changes between surgery and control groups were 0.41 diopters per year (95% CI 0.21 to 0.61; *P* < .001) and −0.17 mm per year (95% CI −0.22 to −0.11; *P* < .001). Subgroup analysis showed significant treatment effects of the single wide strip operation. Single-arm meta-analysis showed less annual axial elongation in children subgroup. These results were robust by sensitivity analysis. The incidence of some major complications in the operation group were significantly greater (5.8% vs 2.7% for myopic degeneration; 2.3% vs 1.6% for macular hemorrhage; 0.8% vs 0 for retinal detachment).

**Conclusion:**

Posterior scleral reinforcement may be an effective surgery on controlling myopia progression by slowing both refraction and axial length change. However, frequent surgical complications should be considered. Further well-designed studies are needed to determine the long-term safety and efficacy.

## Introduction

**Fig 1:**
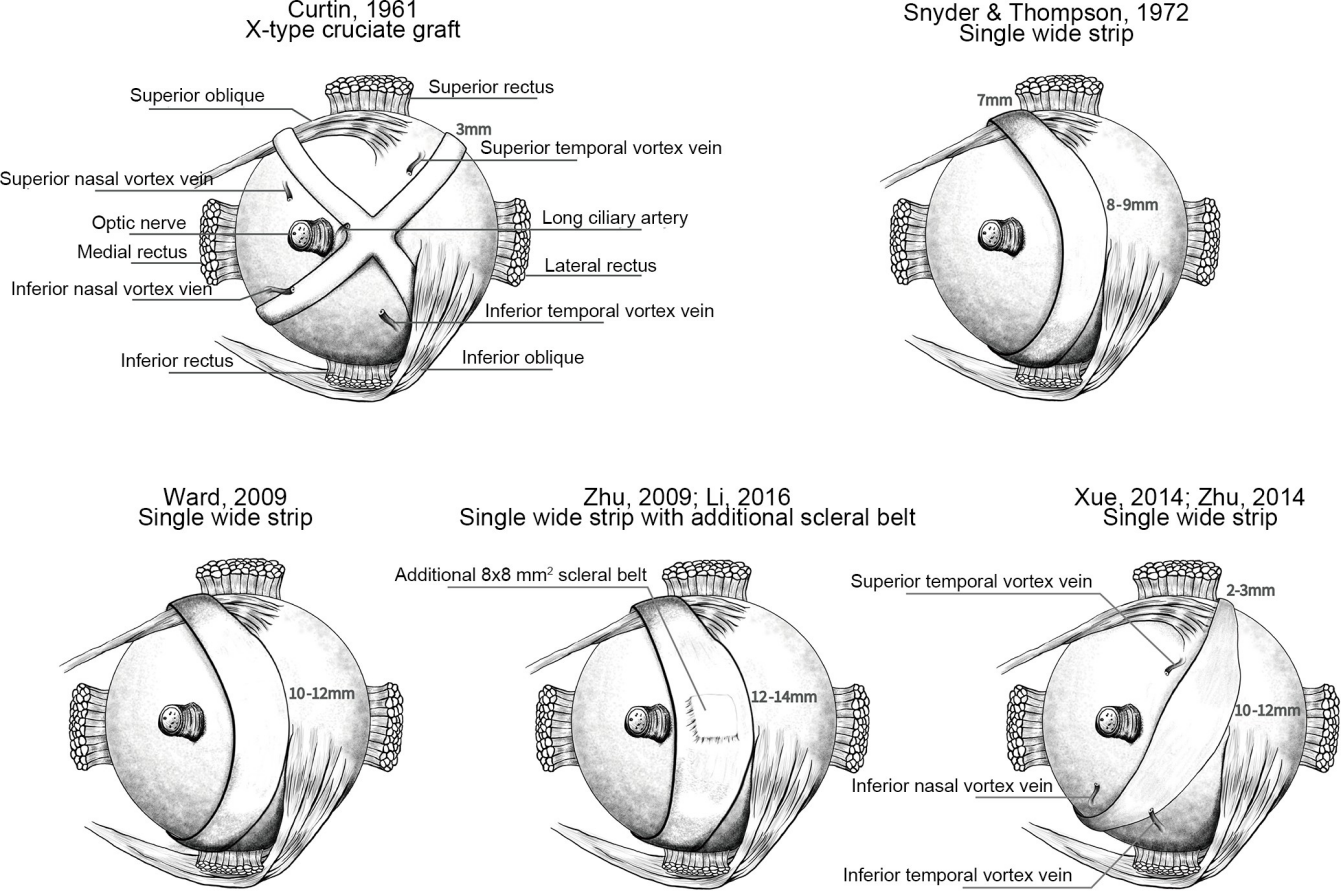
Schematics of the posterior scleral buckle on the right eye from posterior view.

Pathologic myopia is one of the major causes of blindness worldwide. It is an important and sight-threatening disease that causes scleral thinning, axial elongation, localized posterior scleral ectasia, [1] and eventually many severe complications such as retinal detachment, myopic choroidal neovascularization, macular schisis, and macular hole, and macular degeneration. [2] For controlling myopia progression, posterior scleral reinforcement (PSR) surgery was first described by Shevelev in 1930, and was modified as the Snyder-Thompson method in 1972, which is the most common performed operation currently. A graft was held over the macular area by the insertion of the inferior oblique muscle without suturing at the posterior pole, and the two ends of the graft were sutured to the superior and inferior nasal quadrants. In 1961, Curtin described the X-type operation using a cruciate-shaped fascia lata graft extending from the four quadrants of eyeball. The cruciate graft was designed to support a greater area over posterior pole without interposing any muscle or tendon. However, the treatment effect of X-type operation was doubted due to unfavorable long-term outcomes [3] and there were severe complications of cilioretinal artery occlusion, [4] optic nerve pressing and subsequent optic nerve atrophy [5] reported. There were some modifications of the single wide strip PSR. A wider graft was used to support the posterior pole, with the central part as 10 to 12 mm width. [6] To support the posterior pole staphyloma, an additional 8x8 mm^2^ scleral belt was placed between the graft and the eyeball to support the macular area. [2, 7] To form a U-shaped scleral buckle, a spindle-shaped graft was fixed on the two ends at the superior temporal and inferior nasal quadrants. [8, 9] Schematic figures of the different operation methods were shown in Fig 1. Several types of materials have been used as the scleral buckle, including autologous or homologous fascia lata, human sclera, dura, and bovine pericardium. To date, the therapeutic benefits of PSR remain controversial due to a lack of controlled studies investigating the effectiveness. To the best of our knowledge, no systematic review study confirmed or quantitatively defined the treatment effect of PSR operation. Thus, the present study primarily aimed to systematically synthesize all available studies and quantify the efficacy in controlling axial elongation and refraction progression using meta-analysis.

## Materials and methods

### Search strategy

This meta-analysis was conducted in accordance with PRISMA guidelines (S1 Table). [10] We performed a broad and comprehensive literature search from Pubmed, EMBase, and Ovid for studies published from the date of inception to July 24, 2019. We searched for the references of included studies. The search terms we used was in the supporting information, including ‘myopia’, ‘scleral buckle’, ‘posterior sclera* reinforce*’, ‘scleroplasty’, ‘Snyder Thompson’, and ‘buckle reinforce*’. The full search strategy was shown in S2 Table. Only studies in English were included.

### Study inclusion

Two reviewers (C.A.C. and P.C.W.) independently read titles and abstracts to identify possible eligible articles, and then assessed the full text for inclusion. Studies were included according to the inclusion criteria: (1) comparison study including randomized clinical trials (RCT), cohorts, or non-RCT design studies, (2) the participants had myopia without retinoschisis, macular hole, or retinal detachment, (3) studies that estimate the effect of PSR operation on controlling myopia progression, (4) the studies reported outcomes of spherical equivalent refraction (SER) or axial length (AXL). The exclusion criteria were: (1) repeats, (2) the articles failed to report relevant data to calculate the values, (3) non-human studies, (4) review, letters, or comments.

### Data collection

Both reviewers independently extracted data from the included articles for the author, publication year, study design, country, patient ages, sample size, intervention and control methods, follow-up duration, outcomes (change in SER and AXL) and corresponding 95% confidence interval (CI) and/or standard deviation (SD), and number of adverse effects. For postoperative complications analysis, studies with defined specific complication items were included. The complications were grouped into 2 categories, as minor and major sight-threatening complications, including conjunctiva congestion, extraocular movement (EOM) limitation, intraocular pressure (IOP) elevation, choroidal effusion, diplopia, anterior uveitis, myopic degeneration, macular hemorrhage, macular hole, and retinal detachment. Incidence of each specific complication was calculated as the number of eyes with the particular complication over the total number of eyes within the selected studies. We imputed missing data by estimating the covariance (*Cov*) and correlation coefficient (*r*) reported by other studies in this meta-analysis, according to the Cochrane Handbook for Systematic Reviews of Interventions. Sensitivity analysis was performed by imputing with an individual *r* from lower to upper limit each time.

### Quality assessment

For the included studies, the methodological quality was appraised using the Newcastle Ottawa Scale (NOS) for cohort studies. The Cochrane Risk of Bias Tool was applied for RCTs. Disagreements over quality assessment were resolved by discussion, or adjudicated by a third author (P.Y.L.). In addition, we applied funnel plots and Egger’s test to assess publication bias.

### Statistical analysis

Statistical analyses for two-arm and single-arm meta-analyses were performed using Review Manager version 5.3 (Cochrane Collaboration) and Comprehesive Meta-Analysis Software version 3.0 (Englewood, NJ, USA) respectively. In two-arm meta-analysis comparing PSR and control groups, for outcomes in all included studies, myopia progression and axial elongation, defined as change-from-baseline SER and AXL, the weighted mean difference (MD) between cases and controls, corresponding 95% CIs and standard errors were calculated and presented in forest plots. In single-arm meta-analysis, outcomes of the change-from-baseline SER and AXL were obtained from PSR group, and the corresponding 95% CI were calculated. The effect size (ES) was calculated using the Hedges’ *g* to estimate the treatment effect for each outcome. Heterogeneity was assessed using *I*^2^. According to the Cochrane handbook, the heterogeneity was further stratified as follows: 0% to 40% represented heterogeneity as might not be important; 30% to 60% indicated moderate; 50% to 90% represented substantial; and 75% to 100% represented considerable heterogeneity between studies. Sensitivity analysis was performed by disregarding an individual study each time. Subgroup analysis was performed according to different operation methods and age groups to evaluate whether the observed effect size was different across subgroups. The test for subgroup difference in two-arm meta-analysis and the test for total between-groups heterogeneity in single-arm meta-analysis were performed to examine differences among subgroups.

We evaluated the pooled data for meta-analysis with a random-effects model. Statistical significance was set at a two-sided *P* < .05.

## Results

### Literature characteristics

**Fig 2:**
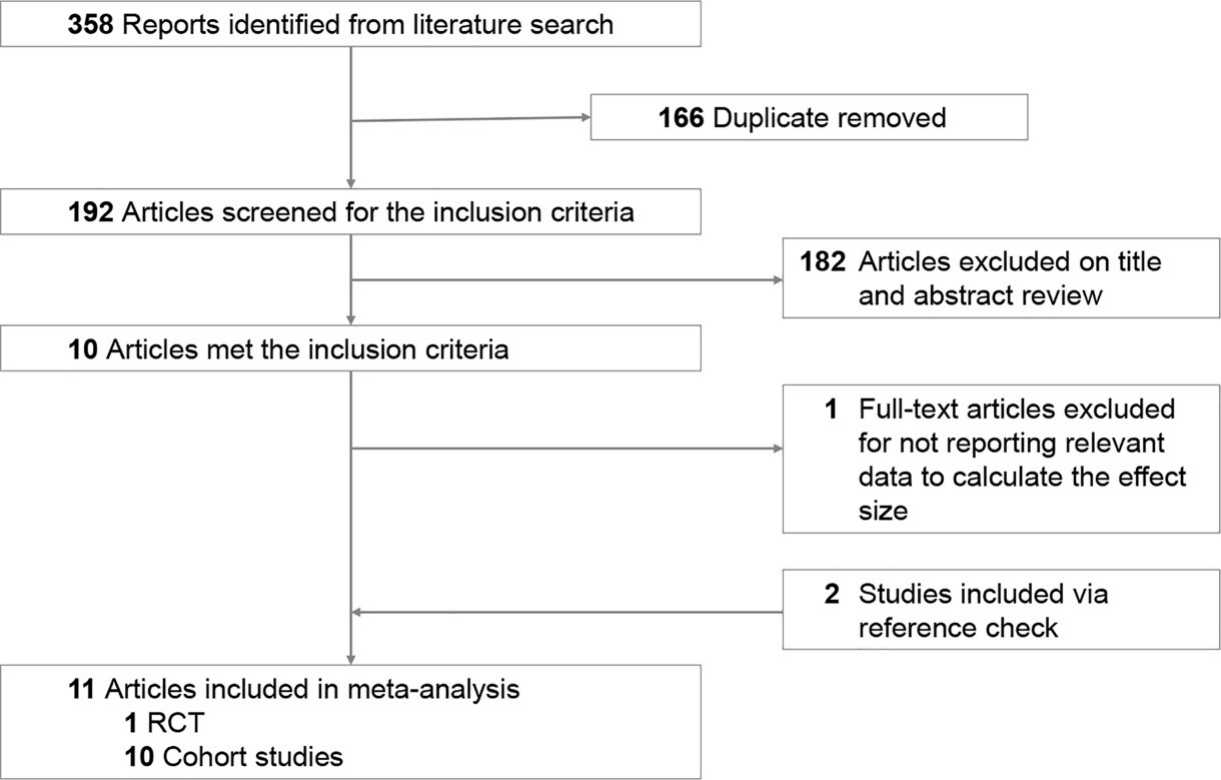
Flow chart of the literature search and study selection.

In this meta-analysis, a total of 358 relevant articles were generated by our search strategy. After removing duplicates, 166 of these articles were retrieved for detailed reviewing of the title and abstract. One hundred eighty-two articles were excluded due to not meeting the inclusion criteria and one article [11] was excluded for not reporting relevant data. Overall, the full text of 9 articles were reviewed to assess eligibility. Two articles were included via self reference check. Finally, our data processing of inclusion and exclusion identified 11 articles in this meta-analysis, including 1 RCT [12] and 10 cohort studies. [1–3, 6, 8, 9, 13–16] The operation methods were single wide strip (n = 9) and X-type PSR (n = 2). Among these studies, 6 studies were self-control studies using the fellow eye as controls, and 5 studies compared PSR versus a control group without PSR treatment. The quality for the cohort studies and the risk of bias for the RCT is shown in S3 and S4 Tables. The data quality of the cohort studies was generally high, with an average score of NOS as 8 (range 7–9). Fig 2 displays the steps of the study inclusion and exclusion process. **Table 1** lists the study characteristics.

**Table 1:** Overview of the studies included in the meta-analysis.

Source	Study Design	Total Participants of Two Arms, PSR/Control, No.	Total Eyes of Two Arms, PSR/Control, No.	Country	Operation Method	Material	Control Method	Mean Age of PSR, y	Mean Age of Control, y	Mean Follow-up, y	BCVA of PSR, Baseline/Post-op, LogMAR	BCVA of Control, Baseline/Follow-up, LogMAR	Baseline Refraction, D	Baseline Axial Length, mm	NOS Score
Chen, [1] 2013	Cohort	41/11	64/17	China	Single wide strip	Human dura	Non-randomized control	6.50±3.23 (2.4–16.4)	7.65±3.61 (1.1–14.3)	5	0.52±0.28/ 0.20±0.15	0.44±0.35/ 0.28±0.25	−10.31±2.45	26.55±1.60	7
Hu, [14] 2018	Cohort	26/23	32/35	China	Single wide strip	Bovine pericardium	Non-randomized control	8.21±3.86	7.28±1.65	1	0.54±0.25/ 0.70±0.22	0.50±0.20/ 0.69±0.21	−11.35±2.38	27.10±1.02	8
Shen, [12] 2015	RCT	16/16	16/16	China	Single wide strip	Human sclera	Randomized control	4.94±0.77 (4–6)	5.06±0.77 (4–6)	3	1.33±0.56/ 0.13±0.14	1.17±0.37/ 0.26±0.12	−11.82±2.88	26.78±1.37	N/A
Xue, [8] 2014	Cohort	35/35	35/35	China	Single wide strip	Human sclera	Self-control	7.5 (4–15)	7.5 (4–15)	2.5	0.24±0.23/ 0.15	0.19±0.21/ 0.11	−9.72±2.79	26.2±1.33	8
Xue, [16] 2018	Cohort	40/40	40/40	China	Single wide strip	Genipin-crosslinked donor scleral strip	Self-control	10 (3–17)	10 (3–17)	2–3	0.20±0.23/ 0.13±0.23	0.15±0.21/ 0.12±0.24	−11.07±3.06	27.60±1.18	8
Zhu, [9] 2014	Cohort	5/5	5/5	China	Single wide strip +PCIOL	Human sclera	Self-control	12.8 (8–17)	12.8 (8–17)	0.25	0.78±0.37 0.33±0.17	0/ 0	−12.9±1.12	27.98±0.71	8
Li, [2] 2016	Cohort	43/36	52/52	China	Single wide strip with additional sclera belt	Human sclera	Non-randomized control	41.03±2.27	40.68±3.32	3	0.52±0.23/	0.51±0.21/	−16.12±2.87	29.49±1.21	8
Peng, [15] 2019	Cohort	26/18	38/30	China	Single wide strip	Human sclera	Non-randomized control	37.36±16.22	36.45±14.21	3	0.33±0.08	0.32±0.11	−15.22±4.62	29.42±1.35	8
Ward, [6] 2009	Cohort	59/29	59/29	US	Single wide strip	Human sclera	Self-control	39 (18–68)	39 (18–68)	3	N/A	N/A	N/A	N/A	9
Curtin, [13] 1961	Cohort	7/7	7/7	US	X-type	Autologous fascia lata	Self-control	8.7 (4–15)	8.7 (4–15)	2	(CF-20/60)/ N/A	(20/200-20/20)/ N/A	−13.29±2.81	N/A	8
Curtin, [3] 1987	Cohort	23/23	23/23	US	X-type	Autologous fascia lata	Self-control	11 (3–51)	11 (3–51)	5	N/A	N/A	−15.48±3.35	28.22±1.79	8

Abbreviation: PSR, posterior scleral reinforcement; BCVA, best-corrected visual acuity; LogMAR, logarithm of the minimum angle of resolution; D, diopter; NOS, Newcastle–Ottawa Scale; RCT, randomized controlled trial; PCIOL, posterior chamber intraocular lens; CF, counting fingers; N/A indicates data not available.

### Refraction progression

**Fig 3:**
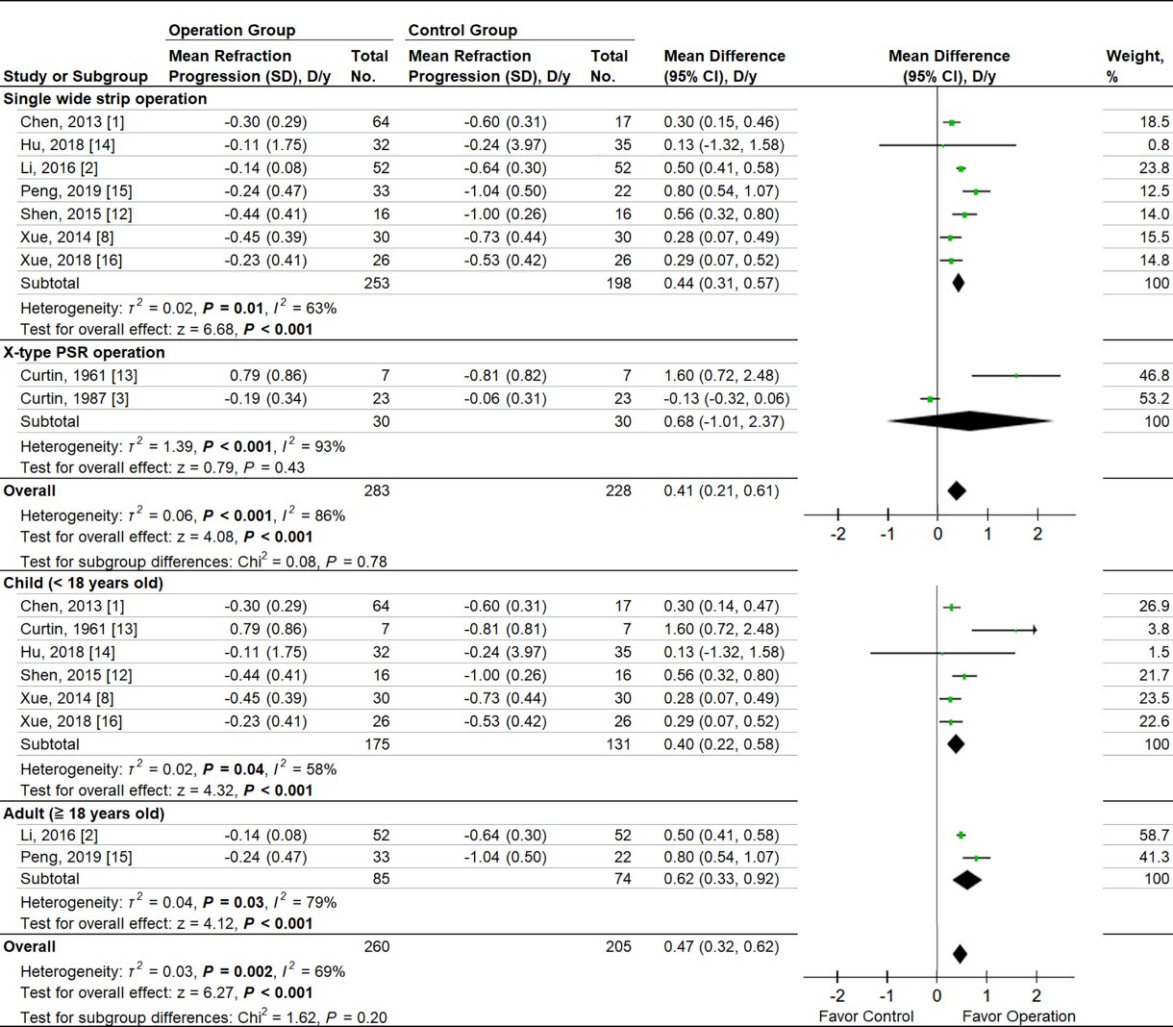
Forest plots of the mean difference in refraction change between therapeutic and control groups with different operation methods, below and above age 18, and the overall treatment effect of PSR operation on refraction. D, diopter; weighted from random effect model.

**Fig 4:**
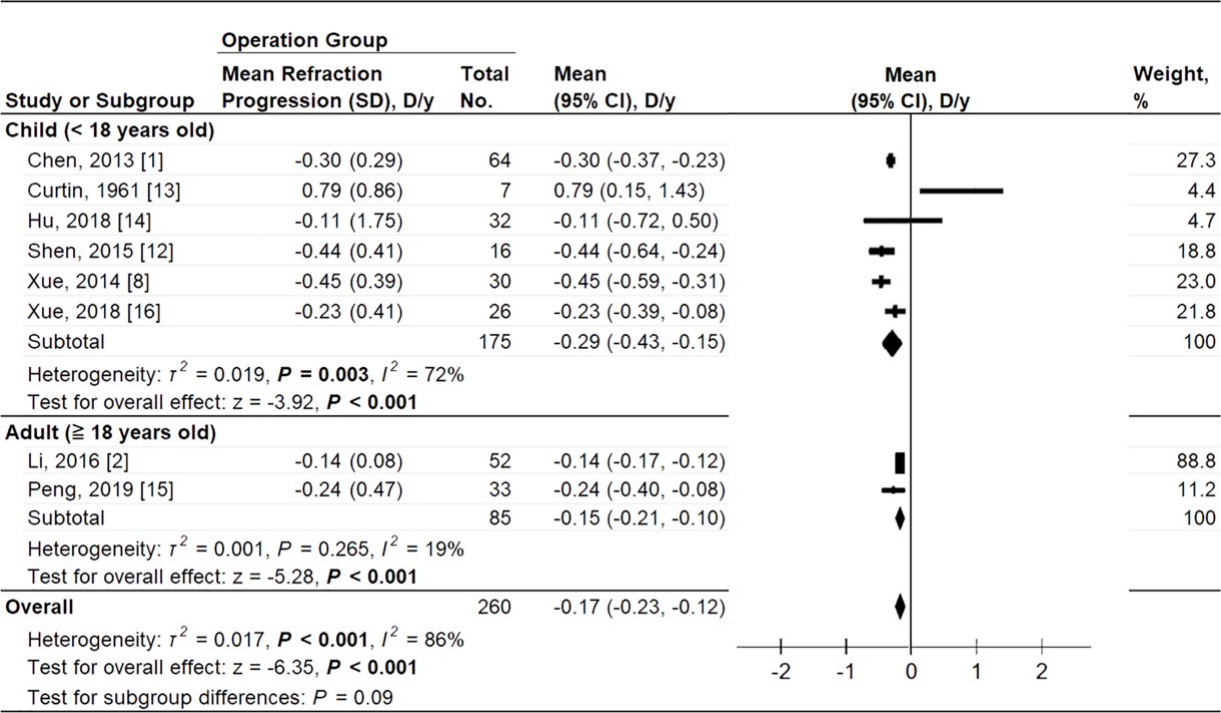
Forest plots of the annual refraction change in the therapeutic group in two different age groups. D, diopter; weighted from random effect model.

One RCT and 9 cohort studies reported data on refraction progression. We pooled RCT and cohort studies. Refraction data from Zhu et al. [9] was excluded due to a significant bias detected by the sensitivity test. The two-arm meta-analysis showed significantly less refraction progression in PSR group (MD = 0.41 diopter per year, 95% CI 0.21 to 0.61; *P* < .001) (Fig 3). The pooling Hedges’ *g* ES was 1.00 (95% CI 0.40 to 1.60; *P* = .001), indicating a large treatment effect. By subgroup analysis, single wide strip was effective on controlling refraction progression but X-type operation was not. A significantly high heterogeneity was noted in the refraction progression when pooling the RCT and cohort studies (*P* < .001, *I*^*2*^ = 86%). The heterogeneity was identified to be associated with different operation methods and age by applying subgroup analysis. Within subgroups, the heterogeneity on refraction progression were significant among single wide strip operation (*P* = .01, *I*^2^ = 63%), X-type operation (*P* < .001, *I*^2^ = 93%), children (*P* = .04, *I*^2^ = 58%) and adult (*P* = .03, *I*^2^ = 79%) subgroups. Test for subgroup difference showed no significant difference in both operation method (*P* = .78) and age subgroup analysis (*P* = .20). In single-arm meta-analysis, the annual SER progression rate in children and adult group showed no significant difference (–0.29 mm/yr, 95% CI –0.43 to –0.15 vs –0.15 mm/yr, 95% CI –0.21 to –0.10; test for subgroup difference, *P* = .09) (Fig 4). The test for subgroup difference showed no significant difference among different age subgroups (*P* = .09).

### Axial elongation

**Fig 5:**
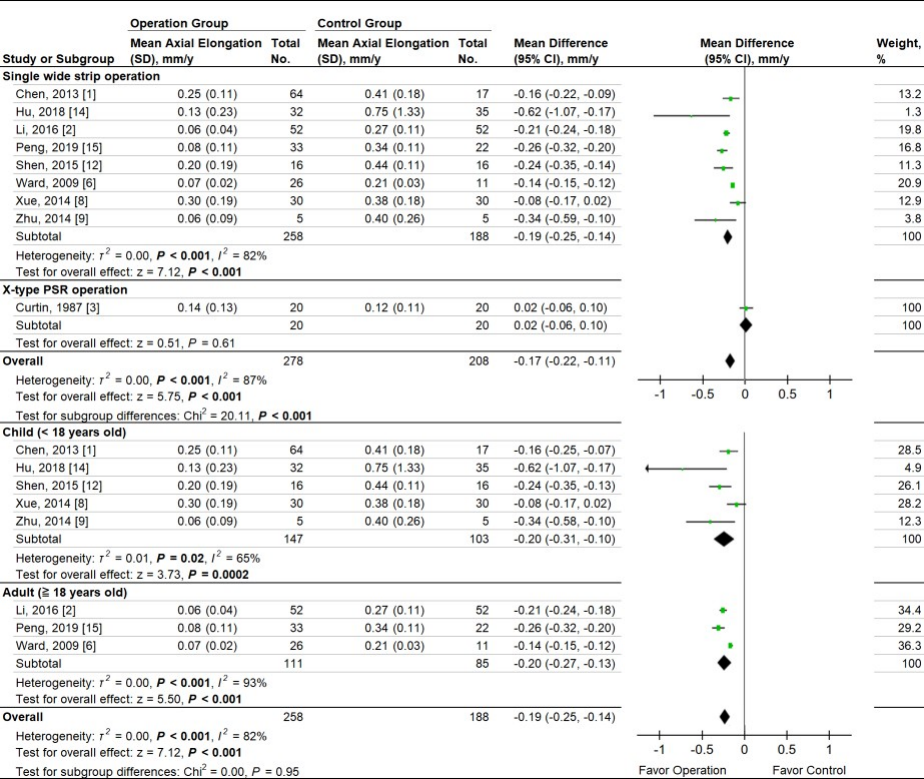
Forest plots of the mean difference in axial elongation between therapeutic and control groups with different operation methods, below and above age 18, and the overall treatment effect of PSR operation on axial length. Weighted from random effect model.

**Fig 6:**
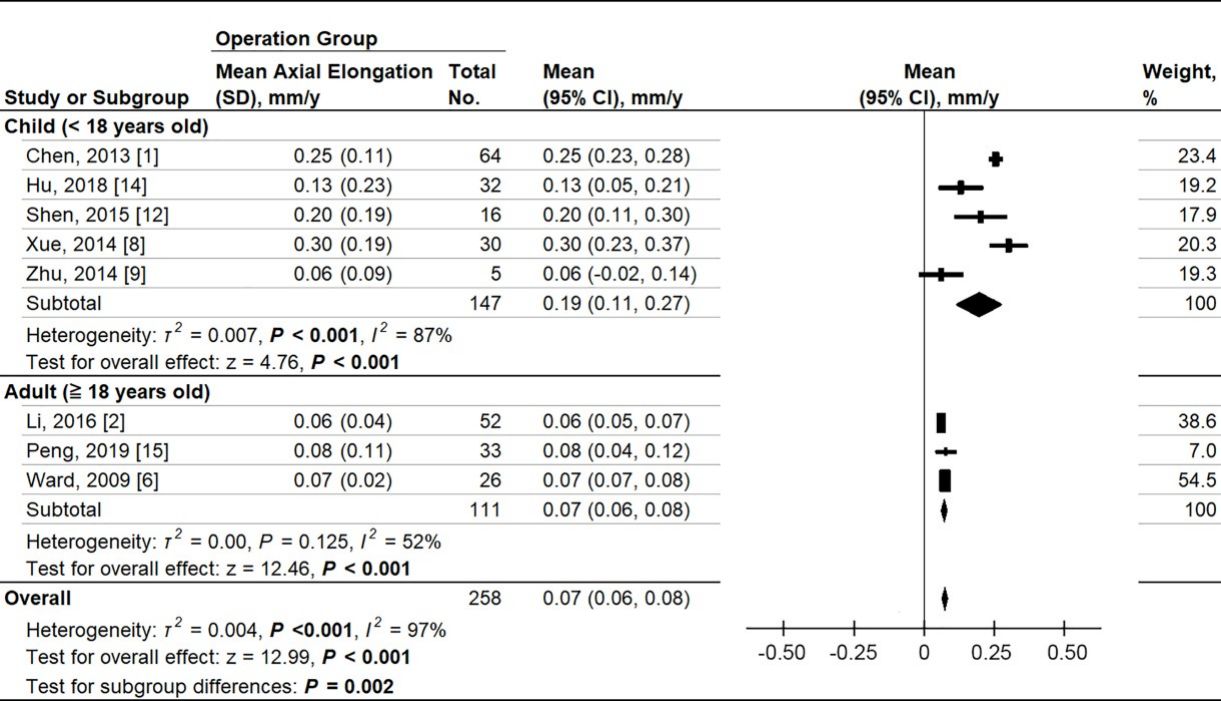
Forest plots of the annual axial elongation in the therapeutic group in two different age groups. D, diopter; weighted from random effect model.

There were 9 studies that reported change-from-baseline AXL between PSR and control groups. Due to a limited number of studies, the RCT and cohort studies were pooled. There was no bias detected by sensitivity test. The two-arm meta-analysis showed that the MD in axial elongation between the PSR and control groups was −0.17 mm per year (95% CI −0.22 to −0.11; *P* < .001) (Fig 5). The pooling Hedges’ *g* ES was −1.65 (95% CI −2.45 to −0.85; *P* < .001). A significant heterogeneity was noted in axial elongation (*P* < .001, *I*^*2*^ = 87%) when pooling the RCT and cohort studies. By operation method subgroup analysis, the single strip operation was effective on controlling axial elongation, but X-type operation was not (test for subgroup difference, *P* < .001). By age subgroup analysis, children and adult subgroups showed no significant difference in treatment effect (*P* = 0.95). In single-arm meta-analysis, the annual AXL elongation rate in children group was significantly faster than adult group (0.19 mm/yr, 95% CI 0.11 to 0.27 vs 0.07 mm/yr, 95% CI 0.06 to 0.08; test for subgroup difference, *P* = .002) (Fig 6).

### Publication bias

Significant publication bias did not present in the funnel plots for refraction progression or for axial elongation.

### Sensitivity analysis

Sensitivity analysis was performed for the treatment effect and heterogeneity. No significant change of treatment effect on refraction progression and axial elongation was observed when omitting an individual study. A cumulative meta-analysis showed similarly robust results. The sensitivity analysis applied for imputing missing data for Li et al. [2] and Shen et al. [12] was stable and reliable, showing no significant difference between calculated ES.

### Adverse effects

Due to limited studies, all RCT and cohort studies were pooled to estimate the incidence of adverse effects. We investigated and quantified the short term and long term sight-threatening adverse effects of PSR. The incidence of surgical complications was reported in **Table 2**. The most common minor complication was conjunctival congestion (99%), followed by EOM limitation (51%), and temporary IOP elevation (21%). One of 151 eyes (0.6%) needed surgical relief for EOM limitation. During the follow up period, some major sight-threatening complications were reported in both PSR and control group, included progression of myopic degeneration (5.8% vs 2.7%), macular hemorrhage (2.3% vs 1.6%), macular hole (0 vs 0.8%), and retinal detachment (0.8% vs 0). The incidence of complication was significantly higher in the PSR operation group.

**Table 2:** The adverse effects for PSR group vs control group.

Complications	No. of studies	Incidence (PSR group vs control)
**Minor**		
Conjunctiva congestion and edema	4	160 of 161 (99%) vs 0
EOM limitation	3	77 of 151 (51%) vs 0
Temporary IOP elevation	6	49 of 233 (21%) vs 0
Shallow choroidal effusion	1	11 of 59 (19%) vs 0
Slight diplopia for 1 month	1	2 of 38 (5%) vs 0
Anterior uveitis	1	1 of 40 (2.5%) vs 0
IOP elevation in 3-year follow-up	6	0 vs 0
**Major sight-threatening**		
Myopic degeneration	4	10 of 172 (5.8%) vs 4 of 147 (2.7%)
Fuchs spot	1	0 vs 2 of 30 (6.7%)
CNV emergence	1	0 vs 1 of 30 (3.3%)
Focal chorioretinal atrophy	2	2 of 75 (2.7%) vs 0
Lacquer crack formation	3	3 of 113 (2.7%) vs 1 of 88 (1.1%)
Progression of posterior staphyloma ectasis	2	5 of 75 (6.7%) vs 0
Macular hemorrhage	3	3 of 132 (2.3%) vs 2 of 124 (1.6%)
Macular hole	3	0 vs 1 of 124 (0.8%)
Retinal detachment	10	3 of 355 (0.8%) vs 0

Abbreviation: PSR, posterior scleral reinforcement; EOM, extraocular movement; IOP, intraocular pressure; CNV, choroidal neovascularization

## Discussion

Our meta-analysis confirmed the PSR operation as an effective treatment for controlling myopia and quantified the treatment effect on refraction progression and axial elongation. Additionally, we investigated and quantified the short term and long term sight-threatening adverse effects of PSR. An early case report described cilioretinal artery occlusion was noted 3 years after receiving PSR. [4] However, there was no cilioretinal artery occlusion noted in our review.

Thompson et al. reported an increasing incidence of post-operative posterior staphyloma and severe macular deterioration when PSR operations were performed on eyes with low-degree myopia. [17] However, in our review, post-operative myopic degeneration was reported in only one study, and those eyes were highly myopic with more than −11.0 diopter before operation. [3] A recent study revealed PSR may maintain the microcirculation of eyes with posterior staphyloma and thereby stabilize the best-corrected visual acuity. [18] The relationship between PSR operation and staphyloma formation remained unclear. Possible mechanisms included the slippage of posterior buckle, and the macula or posterior staphyloma area not covered by the strip, resulting in the further progression of posterior staphyloma from edge of the strip.

The heterogeneity was significantly high when pooling all studies. To investigate the heterogeneity, we subgrouped the studies by operation methods, the mean age, different material of scleral buckle, and ethnicities. The operation subtype was identified as the most important factor affecting the treatment outcome. There were two possible reasons. First, in sensitivity analysis, a significant decrease of heterogeneity among studies was detected when removing Curtin et al. [3] in both refraction progression and axial length elongation and when removing Li et al. [2] in the single wide strip subgroup of axial length elongation. The different operation methods might be the source of heterogeneity since Curtin et al. performed X-type operation, using the cruciate shape periscleral fascia graft, which was thought to support a greater area at posterior pole, [13] whereas Li et al. combined a 12–14 mm single strip buckle and an additional 8*8 mm^2^ sclera belt, which was placed for posterior pole staphyloma in macula. [2] We removed the refraction data of Zhu et al. [9] due to significant heterogeneity detected, which was strongly associated with the operation combining PSR and phakic intraocular lens (PIOL) implantation, causing an extraordinary effect on refraction reduction.

Second, in subgroup analysis, we found that the treatment effect on AXL control of single wide strip operation was better than X-type, with significant subgroup difference (*P* < .001). The X-type operation using a narrow strip, was able to reinforce only macular type posterior staphylomas over a small supporting area of sclera. [3] In contrast, the single wide strip operation not only supported a wider area, but also extended from the superior-nasal to inferior-nasal quadrant, and the inferior staphyloma was thereby reinforced.

In addition, we subgrouped the studies into children and adult groups to investigate the difference of annual axial length increase. We found that there was no significant difference between children and adult groups on the treatment effect of slowing axial elongation. However, single-arm meta-analysis showed in PSR operation group, the annual AXL elongation rate was at least 2.7 times faster in children versus adults (0.19 mm/yr, 95% CI 0.11 to 0.27 vs 0.07 mm/yr, 95% CI 0.06 to 0.08, *P* = .002). A previous study provided the axial elongation curve in myopic children and defined axial stabilization as the annual increases in AXL remained less than 0.06 mm. [19] Our analysis showed that in the children group, PSR operation stabilized axial elongation in only one study by Zhu et al. In contrast, in the adult group, axial length was marginally stabilized (0.07 mm/yr, 95% CI 0.06 to 0.08) by the PSR operation, but the stabilization was not seen in control group. A previous study reported that 11% of myopic children cannot reach axial stabilization even in their adulthood. [19] In our analysis, the non-stabilized adult patients appeared to benefit more from PSR operations than children.

Tideman et al. reported among children between 6 to 9 years of age, the annual AXL elongation for myopic children was 0.34 mm/yr and 0.19 mm/yr for emmetropic children. [20] In our children subgroup, the annual AXL elongation was normalized to 0.19 mm/yr, which was equal to previous reported elongation rate of emmetropic eyes. Preschool-age children had a lesser treatment effect than school-age children after PSR operation. Possible explanations for this observation are that AXL progression is fastest between the ages of 6 and 8 years, [21, 22] and the baseline AXL of pre-school children is much less. Significant heterogeneity within the children subgroup was identified, which could be explained as the rate of eye growth differs with age. Although the PSR operation slows axial elongation, more studies are needed to confirm the treatment effect of PSR operation on children at different ages.

Due to lack of long-term follow up studies for PSR operation, we still cannot give a clear answer that whether the advantages outweighs the disadvantages. In terms of observation group, a 53-year long-term follow-up study showed that in non-selected high myopia cohort, the presence of aging or myopia complicated visual field defect was not correlated to the degree of myopia or extreme AXL. [23] From this we infer that the PSR surgery slows myopia progression anatomically, but functionally, it does not necessarily preserve the visual field, decrease the incidence of myopia complications, or increase the quality of life.

There were some limitations in our study. First, the number of studies included in this meta-analysis was limited. The Eager’s test had limited power to estimate publication bias. Second, some studies failed to report sufficient SD data for calculation, thus we imputed missing data by estimating the covariance (*Cov*) and correlation coefficient (*r*) reported by other studies in this meta-analysis. Despite the limitation, sensitivity analysis for the imputed data showed a robust result. Third, more clinical trials are needed as there was only one RCT was included in our meta-analysis. Fourth, the age and stages of children and corresponding different axial elongation rate may have important influences on the treatment effect. In the children subgroup, we did not stratify the data by age groups due to the children’s ages were not stratified in the included studies. Fifth, considering the significant clinical and methodological heterogeneity among included studies, and the limited number of studies reporting various different complications, it was difficult to assess the complication rate.

In conclusion, this meta-analysis study showed PSR as an effective operation on controlling myopia progression and axial elongation. However, several complications were connected to the operation. Further studies aiming to report the outcomes according to different operation methods and the children’s age groups are needed. Recommendation cannot be made until more randomized prospective cohort study assessing long-term safety and efficacy is available.

## Supporting information

S1 Table PRISMA (preferred reporting items for systematic review and meta-analysis) 2009 checklist. (DOCX)

S2 Table Search strategies. (DOCX)

S3 Table Results of quality assessment using the Newcastle-Ottawa Scale for cohort studies. (DOCX)

S4 Table Results of quality assessment using the Cochrane Collaboration Tool for the RCT. (DOCX)

## References

[1] Chen M, Dai J, Chu R, Qian Y. The efficacy and safety of modified Snyder-Thompson posterior scleral reinforcement in extensive high myopia of Chinese children. Graefes Arch Clin Exp Ophthalmol. 2013;251(11): 2633–8. 10.1007/s00417-013-2429-x 23907482

[2] Li XJ, Yang XP, Li QM, Wang YY, Wang Y, Lyu XB, et al Posterior scleral reinforcement for the treatment of pathological myopia. Int J Ophthalmol. 2016;9(4): 580–4. 10.18240/ijo.2016.04.18 27162733 PMC4853356

[3] Curtin BJ, Whitmore WG. Long-term results of scleral reinforcement surgery. Am J Ophthalmol. 1987;103(4): 544–8. 10.1016/s0002-9394(14)74278-3 3551619

[4] Karabatsas CH, Waldock A, Potts MJ. Cilioretinal artery occlusion following scleral reinforcement surgery. Acta Ophthalmol Scand. 1997;75(3): 316–8. 10.1111/j.1600-0420.1997.tb00784.x 9253985

[5] Momose A. Surgical treatment of myopia—with special references to posterior scleral support operation and radial keratotomy. Indian J Ophthalmol. 1983;31(6): 759–67. 6676262

[6] Ward B, Tarutta EP, Mayer MJ. The efficacy and safety of posterior pole buckles in the control of progressive high myopia. Eye. 2009;23: 2169 10.1038/eye.2008.433 19229272

[7] Zhu Z, Ji X, Zhang J, Ke G. Posterior scleral reinforcement in the treatment of macular retinoschisis in highly myopic patients. Clin Exp Ophthalmol. 2009;37(7): 660–3. 10.1111/j.1442-9071.2009.02111.x 19788661

[8] Xue A, Bao F, Zheng L, Wang Q, Cheng L, Qu J. Posterior scleral reinforcement on progressive high myopic young patients. Optom Vis Sci. 2014;91(4): 412–8. 10.1097/OPX.0000000000000201 24509544

[9] Zhu SQ, Wang QM, Xue AQ, Zheng LY, Su YF, Yu AY. Posterior sclera reinforcement and phakic intraocular lens implantation for highly myopic amblyopia in children: a 3-year follow-up. Eye (Lond). 2014;28(11): 1310–4. 10.1038/eye.2014.200 25125071 PMC4274292

[10] Moher D, Liberati A, Tetzlaff J, Altman DG, Group P. Preferred reporting items for systematic reviews and meta-analyses: the PRISMA statement. PLoS Med. 2009;6(7): e1000097 10.1371/journal.pmed.1000097 19621072 PMC2707599

[11] Coroneo MT, Beaumont JT, Hollows FC. Scleral reinforcement in the treatment of pathologic myopia. Aust N Z J Ophthalmol. 1988;16(4): 317–20. 10.1111/j.1442-9071.1988.tb01234.x 3248182

[12] Shen ZM, Zhang ZY, Zhang LY, Li ZG, Chu RY. Posterior scleral reinforcement combined with patching therapy for pre-school children with unilateral high myopia. Graefes Arch Clin Exp Ophthalmol. 2015;253(8): 1391–5. 10.1007/s00417-015-2963-9 25694153

[13] Curtin BJ. Scleral support of the posterior sclera. II. Clinical results. Am J Ophthalmol. 1961;52: 853–62. 10.1016/0002-9394(61)90328-2 13882758

[14] Hu H, Zhao G, Wu R, Zhong H, Fang M, Deng H. Axial Length/Corneal Radius of Curvature Ratio Assessment of Posterior Sclera Reinforcement for Pathologic Myopia. Ophthalmologica. 2018;239(2–3): 128–32. 10.1159/000484485 29190623

[15] Peng C, Xu J, Ding X, Lu Y, Zhang J, Wang F, et al Effects of posterior scleral reinforcement in pathological myopia: a 3-year follow-up study. Graefes Arch Clin Exp Ophthalmol. 2019;257(3): 607–17. 10.1007/s00417-018-04212-y 30554267

[16] Xue A, Zheng L, Tan G, Wu S, Wu Y, Cheng L, et al Genipin-Crosslinked Donor Sclera for Posterior Scleral Contraction/Reinforcement to Fight Progressive Myopia. Invest Ophthalmol Vis Sci. 2018;59(8): 3564–73. 10.1167/iovs.17-23707 30025077

[17] Thompson FB. A simplified scleral reinforcement technique. Am J Ophthalmol. 1978;86(6): 782–90. 10.1016/0002-9394(78)90121-6 736075

[18] Mo J, Duan AL, Chan SY, Wang XF, Wei WB. Application of optical coherence tomography angiography in assessment of posterior scleral reinforcement for pathologic myopia. Int J Ophthalmol. 2016;9(12): 1761–5. 10.18240/ijo.2016.12.10 28003976 PMC5154989

[19] Hou W, Norton TT, Hyman L, Gwiazda J, Group C. Axial Elongation in Myopic Children and its Association With Myopia Progression in the Correction of Myopia Evaluation Trial. Eye Contact Lens. 2018;44(4): 248–59. 10.1097/ICL.0000000000000505 29923883 PMC6013843

[20] Tideman JWL, Polling JR, Vingerling JR, Jaddoe VWV, Williams C, Guggenheim JA, et al Axial length growth and the risk of developing myopia in European children. Acta Ophthalmol. 2018;96(3): 301–9. 10.1111/aos.13603 29265742 PMC6002955

[21] Zadnik K, Mutti DO, Mitchell GL, Jones LA, Burr D, Moeschberger ML. Normal eye growth in emmetropic schoolchildren. Optom Vis Sci. 2004;81(11): 819–28. 10.1097/01.opx.0000145028.53923.67 15545807

[22] Zhang ZY, Chu RY, Zhang XR, Zhou XT, Hoffman MR, Dai JH. Physical characteristics of ocular structures in Chinese children with emmetropia. J Pediatr Ophthalmol Strabismus. 2011;48(1): 50–6. 10.3928/01913913-20100420-06 20438036

[23] Fledelius HC, Jacobsen N, Li XQ, Goldschmidt E. The Longitudinal Danish High Myopia Study, Cohort 1948: at age 66 years visual ability is only occasionally affected by visual field defects. Acta Ophthalmol. 2019;97(1): 36–43. 10.1111/aos.13820 30284371

