# Supplementary material for: Treatment effect of posterior scleral reinforcement on controlling myopia progression: A systematic review and meta-analysis
Source: PLoS One. 2020 May 26;15(5):e0233564. doi: 10.1371/journal.pone.0233564 (PMC7250442; doi:10.1371/journal.pone.0233564)
Supplement: S2 Table — (DOCX) [file pone.0233564.s002.docx]

**S2 Table. Search strategies**

| **No.** | **Search term** | **No. of results** |
| --- | --- | --- |
| **The following search strategy was used in EMBASE <Search date: 2019/7/24>** | | |
| 1 | ‘myopia’/exp OR myopia | 29625 |
| 2 | ‘scleroplasty’/exp | 17 |
| 3 | ‘scleral buckle’/exp OR ‘posterior scleral reinforcement’ OR (‘sclera*’ NEAR/6 ‘reinforce*’) OR (‘buckl*’ NEAR/6 ‘reinforce*’) OR ‘snyder thompson’ | 557 |
| 4 | #2 OR #3 | 564 |
| 5 | #4 AND #1 | 149 |
| 6 | ‘Macular hole’/exp OR ‘retinoschisis’/exp | 6324 |
| 7 | #5 NOT #6 | 136 |
| 8 | #7 AND [english]/lim | 90 |
| **The following search strategy was used in MEDLINE(Ovid MEDLINE(R) In-Process & Other Non-Indexed Citations and Ovid MEDLINE(R) <Search date: 2019/7/24>** | | |
| 1 | exp Myopia/ | 17178 |
| 2 | exp Scleral Buckling/ | 2776 |
| 3 | Posterior scleral reinforce*.mp. | 31 |
| 4 | buckle reinforce*.mp. | 0 |
| 5 | exp Scleroplasty/ | 79 |
| 6 | ((sclera* adj7 reinforce*) or (buckl* adj7 reinforce*) or snyder thompson).mp. [mp=title, abstract, original title, name of substance word, subject heading word, keyword heading word, protocol supplementary concept word, rare disease supplementary concept word, unique identifier, synonyms] | 121 |
| 7 | 2 or 3 or 4 or 5 or 6 | 2957 |
| 8 | 1 and 7 | 259 |
| 9 | exp Retinoschisis/ or exp Macular Hole/ | 5319 |
| 10 | 8 not 9 | 202 |
| 11 | limit 10 to english | 142 |
| **The following search strategy was used to search Pubmed <Search date: 2019/7/24>** | | |
| 1 | myopia [mesh] | 17192 |
| 2 | scleral buckle [mesh] | 2778 |
| 3 | Posterior[tw] AND sclera*[tw] AND reinforce*[tw] | 54 |
| 4 | buckl*[tw] AND reinforce*[tw] | 85 |
| 5 | scleroplasty [mesh] | 79 |
| 6 | snyder thompson | 727 |
| 7 | #2 OR #3 OR #4 OR #5 OR #6 | 3695 |
| 8 | #7 AND #1 | 238 |
| 9 | macular hole [mesh] OR retinoschisis [mesh] | 5324 |
| 10 | #8 NOT #9 | 181 |
| 11 | #10 AND english [language] | 126 |
