# Supplementary material for: Treatment effect of posterior scleral reinforcement on controlling myopia progression: A systematic review and meta-analysis
Source: PLoS One. 2020 May 26;15(5):e0233564. doi: 10.1371/journal.pone.0233564 (PMC7250442; doi:10.1371/journal.pone.0233564)
Supplement: S3 Table — (DOCX) [file pone.0233564.s003.docx]

**S3 Table. Results of quality assessment using the Newcastle-Ottawa Scale for cohort studies**

|  | **Selection** | | | | **Comparability** | **Outcome** | | |  |
| --- | --- | --- | --- | --- | --- | --- | --- | --- | --- |
|  | Representativeness of the exposed cohort | Selection of the non exposed cohort | Ascertainment of exposure | Demonstration that outcome of interest was not present at start of study | Comparability of cohorts on the basis of the design or analysis | Assessment of outcome | Was follow-up long enough for outcomes to occur | Adequacy of follow up of cohorts | Sum of Score |
| Curtin, 1961 | ★ | ★ | ★ | ★ | ★★ | ★ | - | ★ | 8 |
| Curtin, 1987 | - | ★ | ★ | ★ | ★★ | ★ | ★ | ★ | 8 |
| Ward, 2009 | ★ | ★ | ★ | ★ | ★★ | ★ | ★ | ★ | 9 |
| Chen, 2013 | ★ | - | ★ | ★ | ★ | ★ | ★ | ★ | 7 |
| Zhu, 2014 | ★ | ★ | ★ | ★ | ★★ | ★ | - | ★ | 8 |
| Xue, 2014 | ★ | ★ | ★ | ★ | ★★ | ★ | ★ | - | 8 |
| Li, 2016 | ★ | ★ | ★ | ★ | ★ | ★ | ★ | ★ | 8 |
| Hu, 2018 | ★ | ★ | ★ | ★ | ★★ | ★ | - | ★ | 8 |
| Xue, 2018 | ★ | ★ | ★ | ★ | ★★ | ★ | ★ | - | 8 |
| Peng, 2019 | ★ | ★ | ★ | ★ | ★ | ★ | ★ | ★ | 8 |
