# Supplementary material for: Treatment effect of posterior scleral reinforcement on controlling myopia progression: A systematic review and meta-analysis
Source: PLoS One. 2020 May 26;15(5):e0233564. doi: 10.1371/journal.pone.0233564 (PMC7250442; doi:10.1371/journal.pone.0233564)
Supplement: S4 Table — (DOCX) [file pone.0233564.s004.docx]

**S4 Table. Results of quality assessment using the Cochrane Collaboration Tool for the RCT**

| **Study** | **Randomized sequence generation** | **Allocation concealment** | **Patients and clinician blinding** | **Outcome assess or blinding** | **Adequate follow-up** | **Free from selective outcome reporting** | **Others** |
| --- | --- | --- | --- | --- | --- | --- | --- |
| Shen, 2015 | Low | High | High | Unclear | Low | High | Unclear |
